# Exploring factors influencing the work-related morale for certified nursing assistants in hospice care: A structural equation modeling study

**DOI:** 10.1371/journal.pone.0206281

**Published:** 2018-10-26

**Authors:** Jong-Yi Wang, Hui-Lin Zhuang, Jeng-Yuan Chiou, Chia-Woei Wang, Chen-Yu Wang, Li-Fan Liu

**Affiliations:** 1 Department of Health Services Administration, China Medical University, Taichung, Taiwan; 2 Department of Biological Science and Technology, China Medical University, Taichung, Taiwan; 3 Department of Health Policy and Management, Chung Shan Medical University, Changhua, Taiwan; 4 Department of Obstetrics and Gynecology, Taipei Medical University Hospital, Taipei, Taiwan; 5 Graduate Institute of Clinical Medicine, Taipei Medical University, Taipei, Taiwan; 6 Department of Critical Care Medicine, Taichung Veterans General Hospital, Taichung, Taiwan; 7 Institute of Gerontology, College of Medicine, National Cheng Kung University, Tainan, Taiwan; University of Antwerp, BELGIUM

## Abstract

**Background:**

Certified nursing assistants (CNAs) in palliative care units often encounter the death of patients and undergo emotional burdens because of their altruistic practices. However, the influences of altruism and perceptions of death on the work morale of CNAs remain unclear. The fact that morale can be improved by modifying related factors justifies the necessity of a theoretical model of morale for CNAs. However, such a model is currently absent. Based on relevant literature, factors including altruism, social support, perceptions of death, and job satisfaction are related to morale through the mediation of job stress.

**Aim:**

This study was aimed toward determining the influencing factors and developing a structural model of morale with regard to CNAs working in palliative care units.

**Design:**

The hypothesized model was tested in a cross-sectional design with structural equation modeling using AMOS 23.

**Setting/Participants:**

A total of 413 CNAs from five hospice units in Taiwan participated in the survey.

**Results:**

Goodness-of-fit indexes, including χ2/DF = 2.187, PGFI = 0.461, and PNFI = 0.462, among others, confirmed the modified model fit the data well. Altruism, social support, perceptions of death, and job satisfaction were negatively related to job stress (-0.238, -0.196, -0.204, and -0.131, respectively). Altruism and social support positively affected job satisfaction (0.635 and 0.553). Factors directly affecting morale included altruism, social support, job stress, and job satisfaction (0.837, 0.711, -0.244, and 0.540, respectively). The relationship between altruism and morale was the strongest among all the direct paths toward morale. Job stress was the major mediator in these relationships.

**Conclusion:**

Altruism is imperative for morale. The confirmed model is called the Five Constructs to Morale (5CM) model. These findings indicate the need to assess the levels of altruism and perceptions of death during the recruitment of CNAs. From the perspective of human resource management and organizational behavior, CNAs with a high level of altruism and a positive attitude toward death assure the well-being of end of life patients because of work involvement and commitment to the challenging work of hospice care. The cultivation of a supportive environment and coping strategies are suggested for the management of CNAs facing patient death. Hospice care decision makers should carefully assess morale levels among health professionals by applying the 5CM model. Identifying modifiable factors that can improve morale among nursing staff in hospice care to ensure work fulfillment and quality care merits emphasis in clinical practice. The establishment of effective strategies to facilitate the work morale of nursing staff is suggested for palliative care units. Morale improvement related task should be a global public health priority.

## Introduction

The working conditions in palliative care are demanding and often stressful, which can lead to a state of distress or burnout in health professionals. Previous studies have reported a considerable emotional burden among nurses in intensive care units due to patient death [1]. A highly negative attitude toward death could lead to a professional role conflict and further impact the quality of work in this area. Correspondingly, certified nursing assistants (CNAs), the care workers who have the most contact with end of life (EOL) patients, often encounter death in palliative care units, and such work can be debilitating. Nevertheless, the attitudes CNAs hold toward death and the influences of such attitudes on their work are rarely explored. Whether attitude toward death acts as a determinant of work-related morale in CNAs also remains unclear. Previous studies have indicated that morale is crucial in regard to personnel retention, work fulfillment, and patient satisfaction [2–4]. Hence, investigating job stress and morale among CNAs in palliative care units carries substantial clinical implications for advancing the well-being of both caregivers and receivers.

### Job stress factors in CNAs and theoretical reasoning

The quality of caregivers’ work lives is noted in previous research as a precondition for the provision of quality care to patients. The need for assessing and promoting the quality of life among hospice caregivers has been suggested in prior research [5, 6]. Specifically, the components for measuring the quality of work life include perceived stress, social support, and coping strategies among caregivers [7]. Job stress is a psychological state pertaining to excessive workload and lack of feedback on job performance [8, 9]. Social support, as a predictor of coping strategies, refers to people’s perceptions of the quality of their social relationships [10] that provide environmental resources they may have access to. A previous study indicated that social support and coping strategies are positively related to the quality of work life, whereas stress level has a negative correlation with the quality of work life [11]. The demand-control-support (DCS) model, the most influential theory in occupational health psychology, asserts that levels of work demand (workload), levels of job control, and levels of social support affect the well-being of workers [12]. Lack of support from supervisors and coworkers leads to high levels of job stress in CNAs because of insufficient buffering or coping effects, which has been supported by numerous empirical studies [11, 13–16]. A supportive work environment may assist in ameliorating death-related stress among CNAs [17] and thus reduce work burnout [18, 19]. The DCS model was selected as the theoretical basis because the three factors related to job stress of CNAs, including job satisfaction, altruism, and social support, can be explicated by the three major corresponding components in the DCS model. Therefore, the DCS model provides a relevant theoretical foundation for this study. Moreover, the extant literature has identified a negative relationship between job satisfaction and job stress [8]. Job satisfaction is defined as an overall rating of job characteristics [20, 21] and has been indicated as a potential factor related to job stress.

Regarding the attributes of work in palliative care units, previous studies have indicated that altruistic motives can explain why people are willing to be hospice palliative care volunteers [22, 23]. The values of nursing students have changed over time in a potential trend toward being generally less altruistic, as indicated by extant literature [24], whereas altruism in CNAs requires investigation. Previous studies revealed that job stress might also be influenced by altruism [23, 25] and a positive attitude toward death [1], with negative relationships identified among the two constructs and job stress. In this study, perceptions of death pertain to the fear of death of an EOL patient for whom a CNA is caring and also reflect one’s attitude toward one’s own death [26]. Furthermore, the negative relationship between perceptions of death and job stress found in the aforementioned study could be explained to a large degree by the terror management (TM) theory in a previous study, which states that death-related management functions to “control” the potential for emotional distress through rational and experiential threat-focused defenses [27]. The TM theory posits the human potential to manage terror engendered by the awareness of mortality and is thus relevant to this study in terms of its emphasis on psychological perceptions of death [28]. In a clinical setting, altruism refers to the discretionary practice of concern for the welfare of others in the absence of any external rewards [29] and is the opposite of selfishness [30]. Altruism could be a protector against job stress [23, 25] and has been found to be a significant predictor of volunteers’ length of service at a hospice [31]. Based on the relevant literature, the job stress factors identified among CNAs working in hospice care units were supported by the components of the DCS model and the linkage of TM theory to perceptions of death. Accordingly, altruism, social support, perceptions of death, and job satisfaction were theoretically specified as key factors related to job stress in this study. The extant literature indicated that the four factors could be negatively related to job stress.

### Factors related to morale in CNAs and theoretical reasoning

Previous research has indicated the importance of nursing staff morale [32]. Psycho-social factors that improve morale merit attention. However, a well-established model for morale in CNAs is lacking. Morale has been defined as “a quality of mind and spirit which combines courage, self-discipline, and endurance” [33] and also has been described as an attitudinal response to work conditions that has an impact on the behavior of individuals within an organization [33]. It is often used in conjunction with other attitudinal dimensions, such as satisfaction and commitment. For example, Benge and Hickey [34] made a connection between satisfaction and morale. Furthermore, Vandenberg, Richardson [35] operationalized morale as a latent (unobserved) variable reflecting job satisfaction, organizational commitment, and turnover intention. The theoretical work to date suggests that morale is regarded as a multidimensional construct [36]. To reflect this theoretical complexity adequately, Johnsrud [37] reported that morale represents a comprehensive conception that includes, in addition to work satisfaction, enthusiasm, loyalty, work involvement and commitment, and dedication to common goals. According to the job morale (JM) model proposed by Schaefer and Moos [38], although morale is a motive, the morale of long-term care staff could be affected by work stressors, such as workload. Perceived high levels of workload (demand) and low levels of job satisfaction have been shown to be related to reduced morale. The extant literature has revealed a positive relationship between job stress and turnover intention, with job stress mediating the relationship between job satisfaction and turnover intention [8]; an indirect effect of job satisfaction through the mediation of job stress on turnover intention has also been found. In addition, job satisfaction and job stress could directly and indirectly influence work-related morale [38–42]. These studies, especially testing by path analyses, have indicated that satisfied workers in a work environment with sufficient resources may experience low levels of job stress, which seems to ensure professional motivation. Hence, morale, a more positive concept than turnover, may be changeable, yet improvable. These concepts contributed to the theoretical basis of the hypothesized relationship between job stress and morale in the current study. High levels of job stress may reduce morale. Previous studies have used the intention to stay or quit as an indicator of morale, such as is posited in the JM model. However, this study defines the construct of morale and tests the relationships among these constructs using a more comprehensive approach.

### Theoretical integration and rationale

Extant literature on this topic has indicated that altruism, social support, perceptions of death, and job satisfaction may be related to job stress among hospice caregivers. Furthermore, job satisfaction and job stress as two factors related to turnover intention have been suggested in prior research. Therefore, a total of five constructs that may relate to morale were tested in this study. The relationships among the total of six constructs were theoretically grounded on the DCS model, the JM model, and relevant literature. Altruism may potentially exert motivational control over the stressful job of CNAs when they are facing patient death (the DCS model), whereas job satisfaction that reflects work-related factors (demand) can be linked to job stress and morale (the JM model). The DCS model was used in this study to theoretically describe the factors of job stress among CNAs, whereas the JM model fundamentally suggests that morale can be affected by work-related factors, such as job stress. The two models applied in this study were connected to each other through the overlapped component, job stress, to thus form a theoretical framework (S1 Fig). The idea that morale can be improved by modifying the related factors provides the rationale of this study. Prior research has investigated the effects of morale on work. However, the constructs related to morale are still unknown. Therefore, identifying these factors and improving the morale of CNAs are essential to sustaining the work lives of CNAs in palliative care units.

Because of the significance of morale among CNAs and the dearth of research investigating the relationships among perceptions of death, altruism, and morale, this study was aimed toward testing a structural model of morale among CNAs in palliative care units by using structural equation modeling (SEM), which models concepts as latent variables and can provide research evidence for the relationships, if any, among latent variables [43]. Based on the distinct nature of CNA work in hospice care and also on the extant theories as described by the aforementioned literature, this research hypothesized that altruism, social support, perceptions of death, and job satisfaction are related to morale through the mediation of job stress among CNAs.

## Methods

### Research design

**Fig 1:**
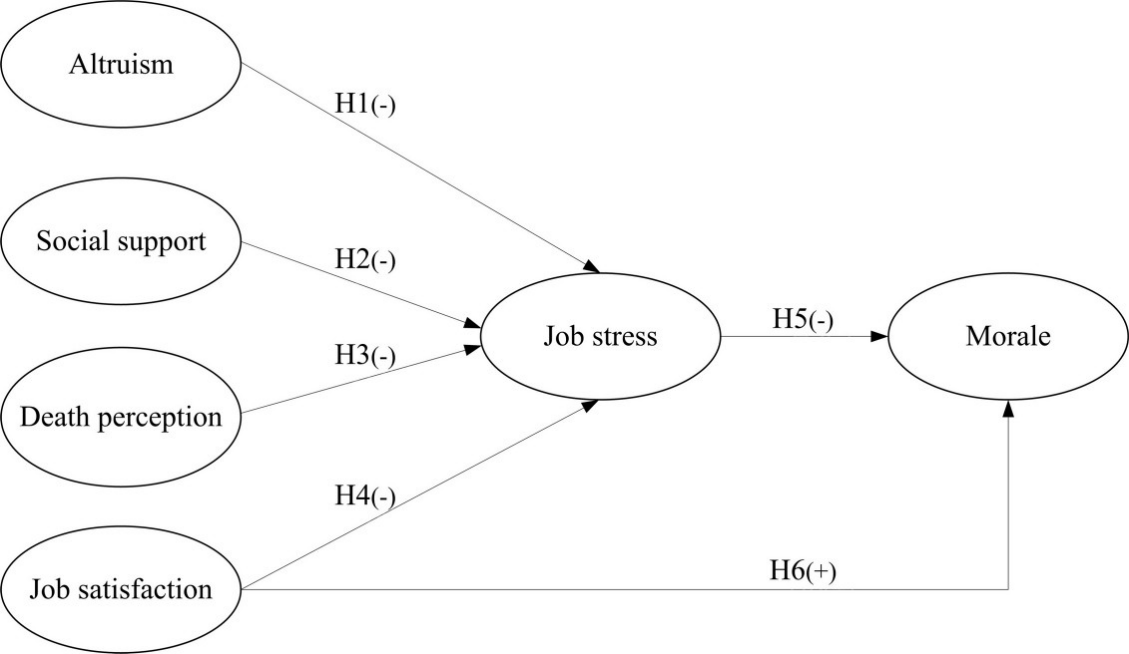
Initial hypothetical model (-: negative relationship, +: positive relationship; major literature that supports the hypothesized paths: H1-[23, 25], H2-[11], H3-[1, 27], H4-[8, 38], H5-[8, 38], H6-[8, 38]).

The theoretical model constructed by this study is presented in Fig 1, which delineates a total of six individual hypothetical relationship paths (H1 to H6) to be tested. All paths were hypothesized to be negative relationships, except the positive relationship (H6) between job satisfaction and morale. The research has been approved by the Institutional Review Boards at China Medical University Hospital (CMUH0-REC3-084), Chung Shan Medical University (CSMUH-CS13226), Taichung Veterans General Hospital (SE-13242), and National Cheng Kung University (B-ER-102-394).

### Participants

A total of 413 CNAs were selected using a mixed sampling method from five hospice care units at five hospitals, including four medical centers and one regional hospital in northern, central, and southern Taiwan. First, although the nursing assistants did not work on shifts, by using a simple random sampling method, one session was selected based on a daytime and nighttime and Monday to Sunday timeframe to fully represent possible fluctuations in mood throughout different sessions. Subsequently, CNAs in the selected session were invited to voluntarily participate in this multi-site study. The time-sampling scheme was repeatedly applied to all five hospice care units across Taiwan. Sample size determination was first calculated by the equations according to the values of related constructs reported by previous studies [39, 44]. The sample size was determined to be sufficient, reaching a satisfactory power of 80%. The proposed research was reviewed and approved by the related institutional review boards. All the participants were asked to sign informed consent forms before filling out the questionnaires, and participation in this survey was completely anonymous. Participating CNAs were asked to complete the questionnaires and notified that they would receive a material reward upon completion of the survey instrument. Consequently, a total of 413 valid CNA samples were obtained, giving a 76.5% response rate. All data were collected from January 2014 to May 2014.

### Research instrument

Six well-established questionnaires were adapted to measure the corresponding constructs. All the items in the questionnaire were scored on a five-point Likert scale ranging from 1 (strongly disagree) to 5 (strongly agree). The initial questionnaire consisted of 39 items measuring the aforementioned six constructs. The questionnaire survey and psychometric properties validation of the research instrument were conducted in two stages. First, a pilot study with a sample of 28 CNAs was performed. Formal administration of the survey was then conducted. The descriptions of the constructs and number of items in the survey are as follows:

1. Altruistic Personality Scale (APS; Rushton, Chrisjohn [45]): Altruism
  APS was adapted to measure altruism. Six items were used for measuring altruism in the survey. After removal of one item for the purposes of the measurement model, the Cronbach’s alpha and factor loadings for altruism were 0.945 and higher than 0.727, respectively. Cronbach’s alpha indicates internal consistency reliability. Factor loadings represent convergent construct validity.
2. Inventory of Socially Supportive Behaviors (ISSB; Barrera, Sandler [46]): Social support
  Items from the ISSB were applied to elicit the participants’ experiences of social support [7, 47]. There were six items used to measure social support in the survey. The Cronbach’s alpha and factor loadings of social support were 0.961 and higher than 0.848, respectively.
3. Collect-Lester Fear of Death Scale (CLFDS; Lester and Abdel-Khalek [26]): Perceptions of death
  Items of the CLFDS were adapted to measure the attitude of CNAs toward dying hospice patients [48]. Perceptions of death as a construct were measured using six items, with a high score representing a positive attitude toward death. After removal of two items, Cronbach’s alpha and factor loadings for perceptions of death were 0.861 and higher than 0.763, respectively.
4. Nurses’ Job Satisfaction (NJS; Mueller and McCloskey [20]): Job satisfaction
  NJS was utilized to assess several dimensions of job characteristics, including the extent to which the CNAs’ work-related needs and rewards were satisfied [20, 21]. Seven items was used to measure job satisfaction. After removal of two items, the Cronbach’s alpha and factor loadings for job satisfaction were 0.859 and higher than 0.478, respectively.
5. Nurse Stress Checklist (NSC; Benoliel, McCorkle [49]): Job stress
  Selected items from the NSC were used in this work to measure the perceived job stress of a CNA. Job stress was measured using seven items, for which the Cronbach’s alpha and factor loadings were 0.870 and higher than 0.489, respectively.
6. Employee Morale Questionnaire (EMQ; Amudha [50]): Morale
  Selected items from the EMQ were revised to assess morale among CNAs. In this study, the concept of morale refers to job identity and involvement and is defined as a psychological quality specifically associated with difficulties [36]. Seven items were used in the survey to measure morale, for which the Cronbach’s alpha and factor loadings were 0.832 and higher than 0.477, respectively.

Three measurements specific to SEM were used in this study, including composite reliability (CR), squared multiple correlation (SMC), and Average Variance Extracted (AVE), to represent the validity and reliability of each construct. CR is the reliability of a unidimensional measurement model for one construct [51]. The CRs of all the constructs were higher than 0.7 (0.89, 0.95, 0.89, 0.74, 0.89, and 0.74 for altruism, social support, perceptions of death, job satisfaction, job stress, and morale, respectively), indicating satisfactory measurements for all the constructs. The SMC analysis demonstrated strong correlations between the observed variables and corresponding latent variables in the measurement model. Furthermore, the values of the AVE indicated that all the latent variables had sufficient convergent validity (> 0.5) and discriminant validity based on the results of the confirmatory factor analysis (CFA) [52].

The Cronbach’s alpha coefficients for all the constructs were also favorable (> 0.8, shown in Table 1). The means of the factor loadings for altruism, social support, perceptions of death, job satisfaction, job stress, and morale were 0.78, 0.87, 0.81, 0.60, 0.70, and 0.56, respectively. No study investigating CNAs by using the same questionnaires was identified based on the current research. However, the values of Cronbach’s alpha for social support and job stress in CNAs (0.96 and 0.87, respectively; Table 1) are similar to those for social support in family caregivers and those for job stress in nurses (0.93 and 0.91, respectively) found in previous studies [53, 54]. The range of factor loadings for social support (0.85 to 0.89) identified in this study is narrower than that (0.34 to 0.86) reported in a previous study for Taiwanese nurses [54]. A principal axis factor analysis with a varimax rotation extracted six corresponding components, accounting for 72.69% of the total variance in the scores for the 34 questionnaire items. Therefore, five questionnaire items were excluded from the former analyses based on the Cronbach’s alpha and factor analysis. After proper removal of the five questionnaire items, all the psychometric properties demonstrated that the questionnaire items comprised an effective instrument for assessing the work-related psychological constructs among CNAs.

**Table 1:** Description of the psychological constructs in the structural model (N = 413).

Constructs	Number of items	Cronbach’s alpha	Factor loadings	Mean	SD
Altruism	5	0.945	> 0.727	3.414	1.175
Social support	6	0.961	> 0.848	3.175	1.049
Perceptions of death	4	0.861	> 0.763	3.021	0.895
Job satisfaction	5	0.859	> 0.478	3.195	0.777
Job stress	7	0.870	> 0.489	2.833	0.758
Morale	7	0.832	> 0.477	3.270	0.933

### Variables

All the questionnaire items, namely observed variables, for measuring the corresponding latent variables, were defined on a five-point Likert scale, ranging from 1 to 5. Altruism, social support, perceptions of death, and job satisfaction were regarded as exogenous variables, whereas job stress and morale were considered endogenous variables. Job stress was treated as the only mediating variable in the hypothetical model. The four exogenous variables acted as the hypothetical protectors of job stress, whereas job stress hypothetically served as the risk factor affecting morale. In addition, hypothetically, job satisfaction functioned as the second direct factor affecting morale. Variables pertaining to the personal characteristics of CNA were defined using either an ordinal or a categorical level and provided supplementary information to that obtained with SEM. Based on the Cronbach’s alpha values and factor loadings of each item during the validity and reliability assessments, one item for altruism, two items for perceptions of death, and two items for job satisfaction were removed from the initial questionnaire to enhance the quality of the measurement. The final numbers of items, Cronbach’s alpha, and factor loadings of the total six latent variables are presented in Table 1.

### Statistical analyses

The main analysis method, SEM, was conducted using AMOS 23. SEM, which is a sophisticated statistical technique suitable for theoretical testing and development in various scientific fields, was used here because of its capacity for measuring error variances in overall theoretical components and their structures. This technique has been also characterized by its ability to estimate multiple and inter-dependent relationships simultaneously and to measure unobserved concepts in these relationships [43]. Therefore, SEM was employed to test the measurement and structural models of all the hypothesized relations among constructs, as depicted in Fig 1. The hypotheses of the theoretical relationships among constructs were determined using SEM. The evaluation for the goodness-of-fit of the hypothesized model included examining the following three sets of criteria: absolute fit measurement, incremental fit measurement, and parsimonious fit measurement [52, 55–57].

Following the commonly used Model Generating (MG) paradigm proposed by Joreskog [58], the current study specified a tentative theory-driven model (Fig 1) and, after the testing, kept the option to modify the model to attain satisfactory goodness-of-fit indexes using the same data. Overall, 34 observed variables extracted to six latent variables were entered into the analysis through SEM.

## Results

In the study, the hypotheses were tested using a cross-sectional design used to analyze the survey data collected from five hospitals in Taiwan. The demographic characteristics of the study sample are presented in Table 2. Most of the CNAs were female (79.90%) and married (61.69%). Approximately one-third of the CNAs were aged 51–60 years (32.58%), had attained a senior high school degree (34.73%), and had 1–3 years of seniority working as a CNA (29.25%). Taoism was the most popular religion in this group (26.54%). Nearly three out of four of the CNAs were Taiwanese (76.23%), with those from Indonesia, Vietnam, and the Philippines accounting for slightly more than one-fifth of those surveyed (20.35%). A majority of the CNAs used both Mandarin and Taiwanese as their major languages (40.10%). The demographic variables, defined categorically, provide additional information to the current study but are not suitable for analysis by SEM, which aims at constructs.

**Table 2:** Characteristics of the samples studied (N = 413).

Variables		N	%
**Gender**			
	Female	326	79.90%
	Male	82	20.10%
**Age (year)**			
	21–30	56	14.04%
	31–40	60	15.04%
	41–50	86	21.55%
	51–60	130	32.58%
	> 60	67	16.79%
**Seniority (year)**			
	< 1	53	13.25%
	1–3	117	29.25%
	4–6	82	20.50%
	7–9	70	17.50%
	≥ 10	78	19.50%
**Marital status**			
	Married	248	61.69%
	Single	154	38.31%
**Education**			
	Elementary school	24	5.91%
	Junior high school	114	28.08%
	Senior high school	141	34.73%
	College or higher	127	31.28%
**Religion**			
	Taoism	108	26.54%
	Buddhism	98	24.08%
	Christianity	79	19.41%
	Islam	12	2.95%
	Other	19	4.67%
	None	91	22.36%
**Nationality**			
	Taiwanese	311	76.23%
	Indonesian	79	19.36%
	Vietnamese	3	0.74%
	Filipino	1	0.25%
	Other	14	3.43%
**Major language**			
	Mandarin	150	36.67%
	Taiwanese	44	10.76%
	Mandarin and Taiwanese	164	40.10%
	Hakka	7	1.71%
	Other	44	10.76%

Table 2 lists the descriptive statistics for all six constructs analyzed in the model. Among all the latent variables measured on a five-point Likert scale, altruism had the highest average score at 3.414, whereas it is notable that job stress was reported at a relatively low average of 2.833. Perceptions of death had an average score in the middle range (Mean = 3.021). Regarding the final construct in the model, while not high, the mean of morale was reported at a moderate level of 3.270.

The modification index (MI), an indicator of a condition used to revise the model in the process of evaluating goodness-of-fit, was used to modify the relational paths among the constructs. When the MI is higher than 5, it is necessary to adjust the model for the residual values [55–58]. Nevertheless, this study used a stricter criterion of an MI higher than 10 to cautiously ascertain that the modification was more theory-based than data-driven.

**Fig 2:**
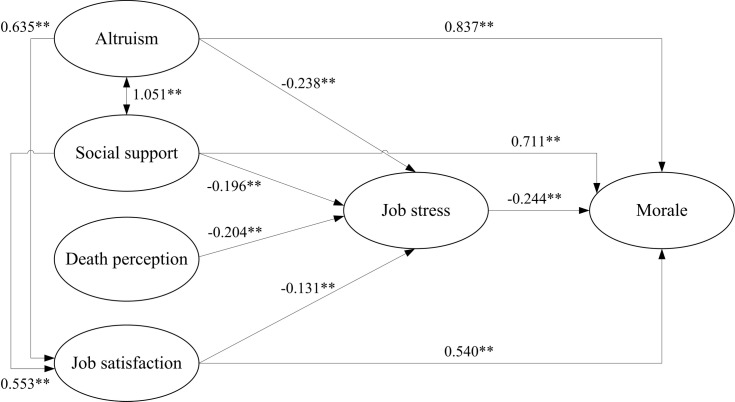
Final model: Five Constructs to Morale (5CM) among CNAs in hospice care (**: *p*-value < 0.001).

All three parsimonious fit measurement indexes showed an adequate model fit, including the essential χ^2^/DF = 2.187, PGFI = 0.461, and PNFI = 0.462. Six additional indexes from the incremental fit measurement and absolute fit measurement confirmed the modified model had a satisfactory fit, including NFI = 0.979, RFI = 0.920, CFI = 0.981, GFI = 0.976, SRMR = 0.049, and RMSEA = 0.079. In short, the final model fit the data well (Fig 2).

According to the final model illustrated in Fig 2, the factors that were directly related to job stress included altruism, social support, perceptions of death, and job satisfaction (-0.238, -0.196, -0.204, and -0.131, respectively; all *p* < 0.001). Altruism and social support were the two constructs that both had a direct relationship (1.051, *p* < 0.001) and also directly affected job satisfaction (0.635 and 0.553, respectively; all *p* < 0.001). Factors directly affecting morale included altruism, social support, job stress, and job satisfaction (0.837, 0.711, -0.244, and 0.540, respectively; all *p* < 0.001). Table 3 lists the direct effects, indirect effects, and total effects among all the constructs. In the ranking of the direct effect size for all 11 significant paths, altruism-social support (1.051), altruism-morale (0.837), and social support-morale (0.711) were the three strongest paths, in that order. All six hypothetical paths from the initial model (Fig 1) were confirmed (Fig 2). In addition, five newly established paths are presented in the final model shown in Fig 2. For example, job satisfaction mediated the relationship between altruism and morale. Because of this mediation result, job satisfaction was identified as an endogenous variable, rather than the original hypothesis suggesting it as an exogenous variable.

**Table 3:** Direct and indirect effects among all the constructs tested in the model (N = 413).

	Direct effect	Indirect effect	Total effect
***Altruism***			
Social support → Altruism	1.051		1.051
***Social support***			
Altruism → Social support	1.051		1.051
***Job satisfaction***			
Altruism → Job satisfaction	0.635		0.635
Social support → Job satisfaction	0.553		0.553
***Job stress***			
Altruism → Job stress	-0.238	-0.083	-0.321
Social support → Job stress	-0.196	-0.072	-0.268
Perceptions of death → Job stress	-0.204		-0.204
Job satisfaction → Job stress	-0.131		-0.131
***Morale***			
Altruism → Morale	0.837	0.058	0.895
Social support → Morale	0.711	0.047	0.758
Perceptions of death → Morale		0.049	0.049
Job satisfaction → Morale	0.540	0.031	0.571
Job stress → Morale	-0.244		-0.244

## Discussion

Improving morale in CNAs, a precondition for the provision of quality care for EOL patients, can benefit both caregivers and receivers in palliative care units through reduced turnover and subsequent improved patient outcomes [4, 59]. Starting from a theoretical construction based on the related literature, this study proceeded to examine a model to explain the relationships among the morale-related constructs by using data from 413 CNAs. The study sample can be characterized by having comparatively high levels of altruism. The mean score for social support in CNAs (3.18, Table 1) was slightly higher than that for family caregivers (3.03) in a previous study [53]. However, the average level of job stress perceived by CNAs (2.83, Table 1) was slightly lower than that by nurses in Taiwan (2.96) [54]. The constructs and their relational paths to morale are identified in the final model (Fig 2), and the implications of the relational paths in the model, as confirmed by SEM, require some interpretation, as follows.

While medical advances may increase survival rates, cancer patients in palliative care units, which were created to offer pain relief and life support, will eventually die. The death of a patient may trigger considerable cognitive, emotional, and behavioral reactions among the CNAs who provide EOL care, including feelings of sadness and frustration [60]. The literature shows that a large proportion (42%) of nursing staff in a long-term care hospital is negatively affected by patient death, suggesting a problem with low morale in this context (H3 and H5) [17]. A previous study revealed that emotional attachment could potentially lead to a greater emotional burden when patient death occurs [1]. Excessive emotional involvement may negatively impact hospice care. The relationship between CNAs’ perceptions of death and their work outcomes warrants intervention. Prior research has indicated that a significant proportion of CNAs (approximately 25%) appear to lack a coping strategy for patient death [17]. Therefore, reasonable expectations regarding death instead of recovery should be established among CNAs who care for people in the last stages of life through education in grief theory [60]. Furthermore, constant monitoring of the emotional health of CNAs and continued education to achieve a greater balance between work involvement and professional quality are essential to the provision of favorable hospice care. Moreover, a supportive work environment may assist in ameliorating death-related stress (H2 and H3) [17]. The finding regarding social support and job stress echoes the stress and coping social support theory [61–63]. Perceived social support may serve to help CNAs cope with stressful events and protect them from the adverse effects of high demand for superior work performance [64]. Therefore, providing a supportive environment to nursing staff who care for patients with serious life-threatening illnesses is crucial.

According to the results of the current study, CNAs displayed a high tendency toward altruism (mean = 3.414), a willingness to act in the interests of others without ulterior motives. The finding that altruism is the most influential motive for hospice care professionals coincides with the results of a previous study [23]. This finding further indicates the direction of recruitment and retention strategies for CNAs [65], which includes identifying candidates whose primary impetus for entering patient care profession is the opportunity to care for others and retaining this motive in CNAs. Various perspectives could be used to interpret individual acts of altruism, including a religious emphasis on achieving unlimited compassion for other human beings, a social norm of mutually beneficial cohesion, a “signal” of an underlying quality based on signaling theory, and a psychological basis due to a certain predisposition to consider others as equals through a shared perception of humanity [29]. The higher levels of morale ascribed to altruism (H1, H5, and a new direct path) could reflect the work-related decisions of individual CNAs who attempt to meet the obligations of social reciprocity and medical practice. Furthermore, altruism as a personality trait may signal to others that the altruist is a cooperative partner who has the ability to obtain resources and evoke trust [66]. From the perspective of the DCS model, highly altruistic motives may thus increase the chance of decision latitude and provide CNAs the equivalent of an intrinsic and autonomous sense of control over their challenging job, which is consequently beneficial to work performance. Although the nature of altruism is multidimensional, the findings of this study provide useful information with regard to using this concept in the recruitment, retention, and possibly the training of CNAs. Hence, regarding the organizational application of this finding to education [67], managers of hospice care units can choose CNAs with high levels of altruism and high morale as role models for their peers in order to promote quality practices [68]. Altruism and job performance in other health care professions also merit more research.

The term “reciprocal suffering” was articulated in previous research into the psychological distress and restrictions on social activities among hospice caregivers that were induced by the constant difficulties that arise due to the nature of their work [5]. However, the difficulties caregivers have in caring for EOL patients could be possibly relieved by implementing “palliative dynamics,” a concept introduced in the existing literature [7] that emphasizes team support and communication among multidisciplinary staff in a palliative care setting. The existing literature has revealed that palliative care work may promote personal enrichment and gratification through making practical contributions to caring for EOL patients. The resulting personal and professional satisfaction may serve as a protective factor against burnout [69]. This finding is similar to the path (H6) identified in this study. Therefore, the development of effective coping strategies, such as emotional support, supervision, and continuing education, is critical to reducing job stress and increasing satisfaction. The study findings warrant an immediate intervention since greater investment in the well-being of nursing personnel leads to better patient outcomes [70].

This study has several findings that should be noted (Fig 2). While some of the relational paths identified in this work are not yet fully supported by the existing literature in the health sector, they are similar to those found in other domains, including the positive relationship between altruism and morale [71, 72], the positive relationship between altruism and social support [73, 74], and the positive relationship between altruism and job satisfaction [75]. Notably, the relationship between altruism and morale was the strongest among all the direct paths toward morale in the model. The influences of social support as a group dynamic on morale and job satisfaction that were found in this work are consistent with those suggested by previous research [76] and should be enhanced in palliative care units. Overall, these findings signal the key role of altruism and social support in morale among CNAs and substantiate the need for additional studies on related topics.

The findings advance related knowledge globally in three major directions. Firstly, altruism functions as the strongest direct factor affecting work morale. Altruism may increase the sense of control over the challenges faced when caring for seriously ill patients. Secondly, perceptions of death that may lead to low morale should be emphasized, especially in EOL care. The fear of death can be managed through proper personal involvement and reasonable expectations of patient outcomes. Thirdly, based on the DCS and JM models, this study further confirmed the mediation of job stress on morale for four related factors. Overall, the current research identified the five modifiable factors affecting morale, including altruism, social support, perceptions of death, job satisfaction, and job stress. The study findings also warrant an intervention for a substantially supportive environment. Identifying modifiable factors that can contribute to improved morale is critical. In the global trend of an aging population, nursing is increasingly important because of the overall impact of nursing practices on health care outcomes, especially in the case of older people [77]. Hence, improved development of nursing services by highlighting and raising the morale of nurses and CNAs should be a public health priority worldwide.

This work has certain limitations that relate to the study sample and the limited use of the questionnaire, as follows. First, this study failed to use a random sampling technique. Although the study sample was selected from five hospitals, the findings should be extrapolated to all other scenarios with caution. Second, 23.77% of the CNAs in this work were not native Mandarin speakers. Even though the researchers who administered the questionnaires did orally explain the items to the non-Mandarin-speaking CNAs when necessary, language comprehension issues could still exist and thus bias the results. Third, all survey scores were based on self-reported measures, thus biasing the findings in an unknown direction. Finally, all data in this study were cross-sectional. No causal effect can be determined from the analysis. Instead, only the relationships can be inferred.

## Conclusion

The present research mainly contributes to existing knowledge by demonstrating the key role of altruism, the distinct influence of perceptions of death, and the mediation of job stress on morale. To the best of our knowledge, this study provides the first research evidence for a structural model of morale among CNAs using SEM in terms of palliative care. The proposed model passed the goodness-of-fit tests carried out using SEM. The resulting confirmed model is called the Five Constructs to Morale (5CM) model. Job stress and job satisfaction are the two major mediators of morale. Different from the existing literature, which investigated morale alone or its effects, this study revealed that morale as a motive could still be affected by other various factors. The 5CM model has both theoretical and clinical implications with regard to advancing the psychophysical well-being of those present at EOL as well as that of the critically ill patients and their caregivers. Future research may apply this model to an investigation of nursing staff using a more comprehensive approach.

The practical implications of the findings include the need to assess altruism and perceptions of death during the recruitment of CNAs for work in palliative care units as well as to provide continued education programs for this profession. The findings also indicate that it is crucial for policymakers to develop psychological/social support programs and coping strategies for CNAs who work in hospice in order to help them better manage patient death. The use of support groups composed of colleagues and supervisors may help to relieve stress in CNAs engendered by the death of patients. From the perspectives of human resource management and organizational behavior, high levels of altruism and positive death attitudes in CNAs will help them with the morale needed to carry out the challenging work of hospice care and thus assure the well-being of EOL patients. Hospice care decision makers can use the 5CM model to assess morale levels among health professionals, such as nurses and CNAs. Identifying modifiable factors related to improved morale that may in turn assure work fulfillment and quality of care should be a priority in clinical practice. The development of effective strategies to advance the work morale of nursing staff is crucial to palliative care units.

## Supporting information

S1 Fig The theoretical framework. (JPG)
