# Supplementary figures and images for: Exploring factors influencing the work-related morale for certified nursing assistants in hospice care: A structural equation modeling study
Source: PLoS One. 2018 Oct 26;13(10):e0206281. doi: 10.1371/journal.pone.0206281 (PMC6203351; doi:10.1371/journal.pone.0206281)

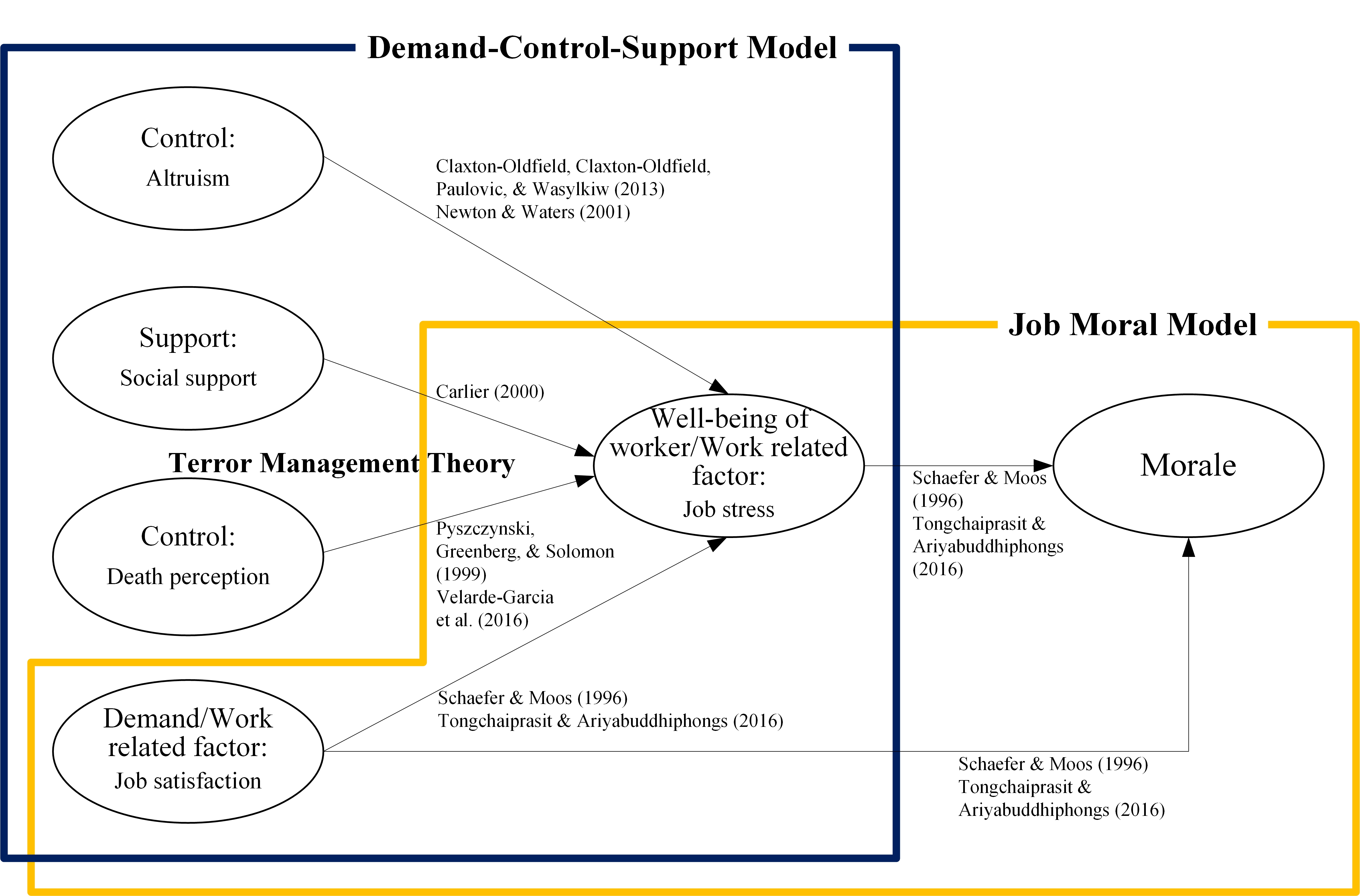

Supplement: S1 Fig — (JPG) [file pone.0206281.s001.jpg]
